# Recognition of chemical entities: combining dictionary-based and grammar-based approaches

**DOI:** 10.1186/1758-2946-7-S1-S10

**Published:** 2015-01-19

**Authors:** Saber A Akhondi, Kristina M Hettne, Eelke van der Horst, Erik M van Mulligen, Jan A Kors

**Affiliations:** 1 Department of Medical Informatics, Erasmus University Medical Center, P.O. Box 2040, Rotterdam, CA 3000, The Netherlands; 2 Department of Human Genetics, Leiden University Medical Center, P.O. Box 9600, Leiden, RC 2300, The Netherlands

**Keywords:** CHEMDNER, BioCreative, Named entity recognition, Chemical dictionaries, Chemical structure, MOL files, Chemical databases, Chemical identifiers, Chemical compounds, Drugs

## Abstract

**Background:**

The past decade has seen an upsurge in the number of publications in chemistry. The ever-swelling volume of available documents makes it increasingly hard to extract relevant new information from such unstructured texts. The BioCreative CHEMDNER challenge invites the development of systems for the automatic recognition of chemicals in text (CEM task) and for ranking the recognized compounds at the document level (CDI task). We investigated an ensemble approach where dictionary-based named entity recognition is used along with grammar-based recognizers to extract compounds from text. We assessed the performance of ten different commercial and publicly available lexical resources using an open source indexing system (Peregrine), in combination with three different chemical compound recognizers and a set of regular expressions to recognize chemical database identifiers. The effect of different stop-word lists, case-sensitivity matching, and use of chunking information was also investigated. We focused on lexical resources that provide chemical structure information. To rank the different compounds found in a text, we used a term confidence score based on the normalized ratio of the term frequencies in chemical and non-chemical journals.

**Results:**

The use of stop-word lists greatly improved the performance of the dictionary-based recognition, but there was no additional benefit from using chunking information. A combination of ChEBI and HMDB as lexical resources, the LeadMine tool for grammar-based recognition, and the regular expressions, outperformed any of the individual systems. On the test set, the F-scores were 77.8% (recall 71.2%, precision 85.8%) for the CEM task and 77.6% (recall 71.7%, precision 84.6%) for the CDI task. Missed terms were mainly due to tokenization issues, poor recognition of formulas, and term conjunctions.

**Conclusions:**

We developed an ensemble system that combines dictionary-based and grammar-based approaches for chemical named entity recognition, outperforming any of the individual systems that we considered. The system is able to provide structure information for most of the compounds that are found. Improved tokenization and better recognition of specific entity types is likely to further improve system performance.

## Background

The past decade has seen a massive increase in the number of chemical publications in the scientific literature. The ever-swelling volume of available documents makes it increasingly hard to manually find and extract relevant information from such texts [1,2]. Automatic indexing of individual publications by the chemical entities mentioned in them, can make it easier to find new information. Ranking these chemical entities by recognition confidence can be helpful in judging the relevance of the publication. Also, knowing the location of every mention of chemical compounds in these publications is of use to establish relationships with other entities or concepts [3].

Different text-mining approaches can be taken to extract chemical named entities from text. The various approaches have been categorized as dictionary-based, morphology-based (or grammar-based), and context-based [3]. In dictionary-based approaches, different matching methods can be used to detect matches of the dictionary terms in the text [3]. This requires good-quality dictionaries. The dictionaries are usually produced from well-known chemical databases. This approach may well capture non-systematic chemical identifiers, such as brand or generic drug names, which are source dependent and are generated at the point of registration. The drawback of a dictionary approach is that it is nearly impossible to also include all systematic chemical identifiers, such as IUPAC names [4] or SMILES [5], which are algorithmically generated based on the structure of the chemical compound and follow a specific grammar [6]. These predefined grammars are sets of rules or guidelines developed to refer to a compound with a unique textual representation (systematic term or identifier). These terms should have a one-to-one correspondence with the structure of the compound. Grammar-based approaches expand their extractions through the capture of systematic terms by utilizing these sets of rules, for example by means of finite state machines [7]. Therefore grammar-based approaches can extract systematic terms that are missing from the dictionaries. Both dictionary-based and grammar-based approaches may suffer from tokenization problems [3]. Following the third approach, context-aware systems use machine learning techniques and natural language processing (NLP) to capture chemical entities. Machine learning techniques utilize the manually annotated chemical terms in a training set of documents to automatically learn and define patterns to extract terms from text [3]. The drawback of machine learning approaches is the need for a sufficiently large annotated corpus for training the system.

Extraction of chemical entities from text has shown to be difficult. Among the main reasons are the large number of terms and synonyms within the chemical domain, the failure to follow guidelines when creating systematic terms by authors, the use of characters such as hyphens and commas within chemical terms, and the ambiguity and inconsistency within and across chemical databases [2,6,8]. Studies have tackled these difficulties using the approaches previously mentioned. Hettne et al. [9] extracted chemical terms from text using a dictionary-based approach (through a system called Peregrine [10]). Funk et al. [11] evaluated the performance of three different dictionary-based systems (MetaMap [12], NCBO Annotator [13], and ConceptMapper [14]) by examining different parameters over multiple ontologies. Lowe et al. developed Opsin, which uses a grammar to transfer chemical nomenclature into structures [15].

In a later study Lowe et al. [16] further improved dictionary-based approaches by introducing 485 grammar-based rules to identify systematic terms. Others (e.g., Leaman et al. [17]) have investigated machine-learning approaches with a focus on conditional random fields (CRFs) [18], hidden mark models (HMMs), and maximum entropy markov models (MEMMs) [19] to extract chemical terms from text. In a recent study, Campos et al. [20] developed Neji, an open source package that integrates dictionary-based and machine-learning approaches to extract biomedical terms from text.

The BioCreative CHEMDNER challenge [8] intends to encourage the development of systems that can index chemical entities (especially the ones that are associated with a chemical structure) in scientific journals. Challenge participants were invited to submit results for two different tasks. The chemical document indexing (CDI) subtask pursues the creation of a list of the chemical entities in a document, ranked according to their confidence of recognition [8]. The chemical entity mention recognition (CEM) subtask aims at establishing the location of every mentioned chemical entity within a document [8]. The CHEMDNER organizers provided the participants with a manually annotated gold standard corpus [21] for training their systems. Overall 65 groups registered for the challenge and 27 groups (both academic and commercial) submitted results [8].

We investigated an ensemble approach where dictionary-based named entity recognition is used along with grammar-based recognizers and chemical toolkits to extract compounds from text. We analyzed the performance of ten different commercial and publicly available lexical resources using Peregrine, an open source indexing system [10,22], along with three different chemical compound recognizers. Different combinations of resources and recognizers were explored to find the best combination to extract the compounds.

## Methods

Our approach was to extract non-systematic chemical identifiers using dictionary-based methods and systematic identifiers using grammar-based methods. We extracted compound family names using a defined ChEBI family dictionary, and database identifiers using a set of manually defined regular expressions. We merged the extractions of these systems. We first concentrated on the CEM subtask where we carried out chemical entity mention recognition. For the CDI subtask we determined confidence scores for all recognized terms and used these to rank the mentions.

### Corpus

The CHEMDNER corpus [21] was used for the development and the evaluation of our system. The corpus consists of 10,000 manually annotated Medline abstracts divided in a training set and a development set (3,500 abstracts each), and a test set (3,000 abstracts). An additional sample dataset with 30 abstracts was also made available through the corpus. The abstracts in the test set were provided as part of a blinded set of 20,000 abstracts (participants did not know which of these abstracts were part of the test set), which the teams had to process in the evaluation phase of the challenge. The corpus has been annotated with the following entity types: abbreviation (e.g., "DMSO"), family (e.g., "Iodopyridazines"), formula (e.g., "(CH3)2SO"), identifier (e.g., "CHEBI:28262"), multiple (e.g., "thieno2,3-d and thieno3,2-d fused oxazin-4-ones"), systematic (e.g., "2-Acetoxybenzoic acid"), trivial (e.g., "Aspirin"), and undefined (e.g., "C4-C-N-PEG9"), concentrating on mentions with practical relevance as to potential target applications (focusing on chemical entities with structures) [21]. Therefore general compounds not associated with chemical structures were not annotated throughout the corpus. The combination of sample set, training set, and development set, collectively called the training material further on, was used to develop the ensemble system.

### Lexical resources

We extracted all the terms (a term denoting a compound and consisting of one or more words) from the databases described below, including brand names, synonyms, trade names, generic names, research codes, Chemical Abstracts Service (CAS) numbers, and any other compound-relevant information. Since we wanted to focus on compounds with structures, only records with MOL file representations of chemical structures [23] were extracted.

#### ChEBI [24]

Chemical Entities of Biological Interest (ChEBI) is a freely accessible dictionary of small molecular entities. Manually checked and annotated (three star) compounds and their associated MOL file representations of chemical structures were extracted, including all synonyms, brand names, ChEBI names, and International Nonproprietary Names (INNs).

#### ChEMBL [25]

ChEMBL is a freely accessible database of bioactive molecules with drug-like properties. Chemical records are manually curated and standardized. Relevant information was extracted from ChEMBL records with associated MOL files.

#### ChemSpider [26]

The ChemSpider database is a freely accessible chemical structure database, owned by the Royal Society of Chemistry [27]. It contains structures, properties and associated information for compounds gathered from more than 470 data sources. The information in the database is validated automatically by robot software, and manually by annotators and crowdsourcing [26,28,29]. We only used the subset of compounds that were manually validated.

#### DrugBank [30]

DrugBank is a freely accessible database containing information on drugs and drug targets. Most of the data in DrugBank is expertly curated from primary literature sources [31]. All synonyms, brand names, CAS numbers, INNs, and generic names were extracted from DrugBank records with MOL files.

#### HMDB [32]

The Human Metabolome Database (HMDB) contains human body-related small molecule metabolites information. The database links chemical, clinical and biological data. All compounds within HMDB are manually annotated by at least two annotators [33].

#### NPC [34]

NIH Chemical Genomics Center Pharmaceutical Collection (NPC) contains clinical approved drugs from the USA, Europe, Canada and Japan. The data are automatically screened for curation [34]. The NPC browser 1.1.0 was used to extract synonyms, CAS numbers, and structure names for compounds with structures.

#### TTD [35]

Therapeutic Target Database (TTD) contains known and explored therapeutic targets and their corresponding drugs. Targets are only included in TTD if they have been described in the literature [36]. All synonyms and drug names were extracted.

#### PubChem [37]

PubChem is a database that provides information regarding biological activities of small molecules. PubChem stores molecular structures and bioassay data from different contributors [37]. A subset of compounds likely to have structure-activity relationships and/or other biological annotations [38] with all of their corresponding synonyms derived from PubChem substances were downloaded.

In addition to the databases above, which all contain information on compound structure, we also explored two large lexical resources that do not provide structure information.

#### Jochem [9]

The joined lexical resource Jochem is a dictionary of small molecules and drugs, containing information from multiple sources. The dictionary is designed for text mining and all integrated data have been filtered, curated and disambiguated automatically [9]. All compounds and their corresponding information were extracted from Jochem.

#### UMLS [39]

The Unified Medical Language System (UMLS) is a collection of biomedical concepts from different lexical resources grouped by 135 different semantic types [39]. UMLS provides a mapping among these lexical resources. Automatic auditing tools are used to discover and resolve possible errors [40,41]. Concepts belonging to a subset of 21 chemical-related semantic types were selected and extracted from UMLS.

To capture family names, we also created a dictionary from the ChEBI ontology where we only took parent compounds that did not appear in the ChEBI three-star database, assuming that these terms have a high likelihood of being a family name. We call this dictionary **ChEBI family**.

Table 1 shows the number of compounds and the number of terms for each of the resources. The total number of unique (case-sensitive) terms was 25,795,580.

**Table 1:** Number of records and number of terms in the terminological resources.

Resource	Number of compounds	Number of terms	Structure
ChEBI	23,240	85,036	Yes
ChEMBL	22,245	29,488	Yes
ChemSpider	2,957,105	5,235,393	Yes
DrugBank	6516	31,991	Yes
HMDB	40,200	364,541	Yes
NPC	14,666	131,795	Yes
TTD	3,196	127,568	Yes
PubChem	4,235,189	19,420,462	Yes
Jochem	362,928	2,062,333	No
UMLS	329,464	743,791	No
ChEBI Family	22,635	90,166	No

### Stop words

In a recent study, Funk et al. [11] described the effect of different parameters such as use of stop words on automatic extraction of biomedical concepts from text. In this study we investigate the influence of stop words on automatic extraction of chemical terms from text. Several stop-word lists were analyzed for their ability to improve system performance, viz. English basic words (100 words) [42], the PubMed stop-word list (133 words) [43], the Jochem stop-word list (258 words) [9], and stop-words derived from the CHEMDNER annotation guidelines (116 words) [21]. Terms found by dictionary-based or grammar-based matching were disregarded if they were part of the stop-word lists. The basic English stop-word list and the PubMed stop-word list contain common English words, with 51 shared terms like "about", "all", "most", and "make". The Jochem stop-word list and the CHEMDNER derived stop-word list focused on more specific ambiguous terms, such as "crystal" or "acid" for the Jochem set, and "insulin" or "lead" for the CHEMDNER set. These two sets only shared five words.

### Dictionary-based recognition

We employed the Peregrine tagger [10,22] to analyze the performance of the individual terminological resources. Tokenization of text that contains chemical terms can be complicated as compound names may include punctuation, such as commas or brackets. We used Peregrine with the tokenizer previously developed by Hettne et al. [9]. All the terms from the terminological resources were used to index the training material with different settings for case sensitivity and noun-phrase (NP) chunking.

#### Case sensitivity

To study the effect of case sensitivity of characters within chemical names on the performance of the system, we indexed the text in separate runs with different matching settings: case insensitive, case sensitive, and partial case sensitive (only case sensitive for abbreviations, defined as terms where the majority of characters consists of capitals and digits, e.g. "BaTiO3").

#### NP chunking

Assuming that chemical compounds will mostly be present in the noun phrases of a sentence, the experiments were also repeated by only feeding noun phrases extracted with the OpenNLP chunker [44] to Peregrine. The OpenNLP chunker has previously been shown to score best in performance and usability on NP recognition in biomedical text [45].

### Grammar-based recognition

A number of public and commercial software packages that can find chemical entities in text were used for the grammar-based recognition approach. ChemAxon's Document-to-Structure toolkit (D2S) [46], NextMove's LeadMine [47], and OSCAR 4 [48] were used for this purpose. These tools have also implemented grammar-based recognition of systematic chemical identifiers. D2S uses grammars along with dictionaries to extract chemicals from text. D2S can also extract information from optical character recognition text and has the ability to recognize chemical structures from text (image extraction) [46]. NextMove's LeadMine uses a filtered dictionary along with 485 rules (grammars defined for chemical nomenclatures naming) to find and extract systematic names. The tool provides automatic spelling correction which allows the tool to extract misspelled terms from documents. The tool also supports multiple languages [47]. Oscar is an open-source software package for extracting named entities from chemical publications. The tool uses different types of models (such as a Bayesian model, pattern recognition, and a Maximum Entropy Markov Model) to extract terms from documents [48]. All the tools were used with their default settings, without further training, adjustment or tuning.

### Regular expressions

Database identifiers of compounds are one of the entity types annotated in the CHEMDNER corpus [21], e.g., LY541850 or AMN082. This subset was used to define a set of regular expressions that served to index the abstracts for chemical database identifiers. As an example, "LY[\ ]{0,1}[1-9][0-9]{5,6}" captures the letters "LY" followed by a space (optional) and six or seven digits (the first of which is not 0).

### Ensemble system

The stop-word lists were employed for both dictionary-based and grammar-based recognition. The dictionary-based recognition was applied using different settings for case sensitivity and NP chunking. We used the BioCreative evaluation script [49] to calculate precision, recall, and F-score (using exact matching of entity boundaries without considering entity type). The scores for the grammar-based recognizers and the regular expressions were also calculated in the same manner. We then heuristically selected different combinations of terminological resources, grammar-based recognizers and regular expressions, and assessed the performance of each ensemble. Our strategy was to have at least one system from each approach. The ensemble system merged the outputs of the various systems. All combinations of up to three lexical resources, the grammar-based recognizers, and the regular expressions were assessed, and the ensemble system with the highest F-score was determined. For comparison, we also investigated a simple voting scheme, where a term is accepted if the number of resources and systems by which the term is found, is at least equal to a voting threshold.

In the final setup we tried to improve our system by extending our dictionary with all gold-standard annotations from the training material that our system initially missed. Further improvement was reached by singling out indexed terms that overlapped. In these cases, the longest term (greater number of characters) was kept. If the terms had the same number of characters, they were ranked based on the subsystems that extracted them: regular expressions, grammar-based, dictionary-based (decreasing priority). If any or both of the overlapping terms were captured by more than one system, the term with highest priority was chosen. In rare cases where the overlapping terms had the same size and the same priority, one term was randomly chosen.

### Ranking

To perform the CDI subtask, we needed a sorted list of unique mentions of the chemical terms in each document. The terms should be ranked according to an estimated confidence of recognition. We therefore determined a "confidence score" for each chemical term as follows. Abstracts from the whole of Medline were divided into two subgroups based on subject categories from the ISI Web of Knowledge [50] (Table 2). The first group consisted of 1,979,485 abstracts from chemical journals, employing the same subject categories as described in the CHEMDNER guidelines [21]. The second group contained 73,603 abstracts from non-chemical journals (e.g., journals in the subject category "Agricultural economics & policy") carefully chosen through the ISI Web of Knowledge classification. All abstracts were indexed by Peregrine with all lexical resources. We assumed that chemical terms would be present more frequently in chemical abstracts than in non-chemical abstracts. For each term, the ratio of the tf*idf (term frequency times inverse document frequency) scores for both abstract sets was computed and transformed into a confidence score between zero and one: if ratio < 1 then score = ratio * 0.5 else score = 1 - 0.5/ratio. A term with high confidence is found more frequently in chemical abstracts than in non-chemical abstracts and therefore is likely to be a chemical term. Vice versa, a term with low confidence is likely to be non-chemical, or highly ambiguous. For example, the drug "Indomethacin" (with DrugBank id DB00328) was found 15,421 times in the chemical abstracts and only once in the non-chemical abstracts, resulting in a high confidence score of 0.99. The ambiguous term "Merit" (synonym of "Imidacloprid" with HMDB id HMDB40292) was found 779 times in the chemical and 101 times in the non-chemical abstracts and obtained the low score of 0.14 after normalization.

**Table 2:** Subject categories in the ISI Web of Knowledge that contain chemical or non-chemical related journals.

Chemical related	Non-chemical related
Biochemistry & molecular	Agricultural economics & policy
Biology	Automation & control systems
Chemistry, applied	Computer science, information systems
Chemistry, medicinal	Computer science, software engineering
Chemistry, multidisciplinary	Computer science, theory & methods
Chemistry, organic	Education, scientific disciplines
Chemistry, physical	Instruments & instrumentation
Endocrinology & metabolism	Mathematics
Engineering, chemical	Mechanics
Polymer science	Physics, mathematical
Pharmacology & pharmacy	Robotics
Toxicology	Telecommunications

The confidence score was taken to rank the term. If it was not available (due to time constraints for the challenge we did not compute scores for terms only captured by regular expressions or grammar-based recognition, which took much more processing time than dictionary-based recognition), the term was ranked according to the precision of the system that indexed the term. In cases where multiple systems indexed the term the highest score was applied.

## Results

### Individual systems

Table 3 shows the baseline performance of the dictionary-based and grammar-based named entity recognition with and without stop-word removal on the 7030 abstracts in the training material. The dictionary-based named entity recognition was performed with case sensitive matching.

**Table 3:** Performance (in %) of individual systems on the training material, before and after stop-word removal.

	Baseline			Baseline + stop-word removal		
		
	Precision	Recall	F-score	Precision	Recall	F-score
Dictionary-based						
ChEBI	28.3	40.6	33.4	77.7	39.7	52.6
ChEMBL	**87.9**	18.7	30.8	**88.8**	18.7	30.9
ChemSpider	65.4	39.0	48.9	80.4	38.4	51.9
DrugBank	63.0	17.2	27.0	78.1	17.1	28.1
HMDB	53.2	34.5	41.8	81.3	33.9	47.9
NPC	46.8	26.7	34.0	59.7	26.4	36.6
TTD	43.9	14.7	22.1	82.9	14.4	24.6
PubChem	17.4	59.0	26.9	61.1	57.9	**59.5**
Jochem	64.2	52.5	**57.8**	67.1	52.5	58.9
UMLS	37.7	51.1	43.4	45.4	50.8	47.9
ChEBI Family	10.4	16.6	12.8	29.4	16.3	21.0
Grammar-based						
Oscar	25.1	**63.2**	35.9	28.4	**62.4**	39.0
LeadMine	64.9	47.4	54.8	74.6	47.1	57.7
ChemAxon	80.9	41.8	55.1	82.5	41.7	55.4

The highest score in each column is bolded.

The baseline F-scores without stop-word removal fluctuate between 12.8% and 57.8%, with Jochem, ChemAxon and LeadMine performing the best. ChEMBL obtained a high precision of 87.9% but with a poor recall of 18.7%. Oscar, PubChem and Jochem had the highest recalls, but with moderate to poor precisions. ChEBI Family gained the lowest F-score, which can be explained by the fact that its scope was limited to chemical family names. Further analysis revealed that 40.3% of the annotated family names were captured by ChEBI Family. The low precision of ChEBI Family is mainly due to the presence of terms such as "role", "proteins", "inhibitors", "metabolites", which are not blocked as they are not present in the stop-word list. The use of the stop-word lists greatly improved the precision and F-score of the majority of resources. The performance of ChEMBL and ChemAxon remained nearly constant showing that these systems extract few of the stop words in our lists. Use of the stop-word lists hardly affects recall, with a largest decrease of only 1.1% for PubChem.

Table 4 gives a further breakdown of the performance improvement for the individual stop-word lists that were used. Clearly, the largest improvements are seen for the Basic English terms (up to 23.7 percentage points with an average of 4.1) and the PubMed stop-word list (up to 22.3 percentage points with an average of 3.6). Among the terms that had a large effect on precision were basic English terms such as "In" (extracted 32367 times of which only 5 are annotated in the corpus as Formula) and "As" (extracted 7087 times of which 33 cases are annotated as Formula). Many more general terms were also extracted mostly as false positives, such as "protein", "DNA", "insulin", and "water".

**Table 4:** Effect of individual stop-word lists on F-score.

	Baseline			Basic English			PubMed stop words			Jochem stop words			CHEMDNER guidelines		
					
Resource	P	R	F	P	R	F	P	R	F	P	R	F	P	R	F
ChEBI	28.3	40.6	33.4	69.0	40.3	50.9	63.0	40.3	49.2	28.5	40.1	33.3	29.3	40.6	34.0
ChEMBL	87.9	18.7	30.8	87.9	18.7	30.8	87.9	18.7	30.8	88.8	18.7	30.9	87.9	18.7	30.8
ChemSpider	65.4	39.0	48.9	74.4	38.8	51.0	65.3	38.8	48.7	69.3	38.6	49.6	67.9	39.0	49.5
DrugBank	63.0	17.2	27.0	63.0	17.2	27.0	63.0	17.2	27.0	78.1	17.1	28.1	63.1	17.2	27.0
HMDB	53.2	34.5	41.8	74.6	34.4	47.1	72.6	34.4	46.7	55.7	34.0	42.2	55.2	34.5	42.5
NPC	46.8	26.7	34.0	47.6	26.5	34.0	46.6	26.5	33.7	52.2	26.7	35.3	53.3	26.7	35.6
TTD	43.9	14.7	22.1	64.9	14.5	23.7	66.0	14.5	23.8	50.6	14.7	22.8	43.9	14.7	22.1
PubChem	17.4	59.0	26.9	44.5	58.7	50.6	42.4	58.6	49.2	18.9	58.3	28.5	17.9	59.0	27.4
Jochem	64.2	52.5	57.8	65.2	52.5	58.2	64.2	52.5	57.8	64.1	52.5	57.7	67.1	52.5	58.9
UMLS	37.7	51.1	43.4	45.4	50.8	43.6	38.0	51.1	43.6	40.0	50.8	44.9	42.4	51.1	46.4
ChEBI	10.4	16.6	12.8	21.0	16.6	18.5	21.0	16.6	18.5	10.8	16.4	13.1	11.6	16.6	13.7
Family Oscar	25.1	63.2	35.9	25.4	63.0	36.2	25.3	62.9	36.1	25.7	62.7	36.4	27.7	63.2	38.5
LeadMine	64.9	47.4	54.8	66.4	47.4	55.3	64.9	47.4	54.8	68.0	47.1	55.7	72.8	47.4	57.4
ChemAxon	80.9	41.8	55.1	80.9	41.8	55.1	80.9	41.8	55.1	81.1	41.7	55.1	83.3	41.8	55.5

### Case sensitivity

To study the influence of case sensitivity on the dictionary-based approach, we indexed the training data using case insensitive, case sensitive, and partial case sensitive matching for all terminological resources (Table 5). The results did not show a large difference in most of the cases although (partial) case sensitive matching improved the F-score of ChEBI by 7.1 percentage points and reduced the score of TTD by 2.7 percentage points.

**Table 5:** F-score of terminological resources for different case sensitivity matching.

	Insensitive			Sensitive			Partial sensitive		
			
Resource	P	R	F	P	R	F	P	R	F
ChEBI	71.2	33.5	45.6	77.7	39.7	52.6	76.7	40.2	52.7
ChEMBL	91.6	18.9	31.3	88.8	18.7	30.9	88.5	18.8	31.1
ChemSpider	78.4	40.5	53.4	80.4	38.4	51.9	80.3	39.6	53.0
DrugBank	76.0	17.5	28.4	78.1	17.1	28.1	78.4	17.5	28.6
HMDB	79.3	35.1	48.6	81.3	33.9	47.9	81.5	35.1	49.1
NPC	58.5	26.8	36.8	59.7	26.4	36.6	59.9	27.1	37.4
TTD	78.3	16.8	27.6	82.9	14.4	24.6	81.1	14.7	24.9
PubChem	56.4	57.2	56.8	61.1	57.9	59.5	60.4	58.6	59.5
Jochem	67.1	52.5	58.9	67.1	52.5	58.9	66.4	53.5	59.3
UMLS	44.7	51.6	47.9	45.4	50.8	47.9	45.3	51.3	48.1
ChEBI Family	29.4	16.3	21.0	29.4	16.3	21.0	29.4	16.4	21.1

### NP chunking

To study the possible gain through NP chunking on dictionary-based approaches, we applied the OpenNLP chunker to extract noun phrases from the training material. The noun phrases were then indexed with Peregrine using all terminological resources. Table 6 shows higher precision and F-scores for most of the systems as compared to the baseline values (cf. Table 3), in particular for PubChem and ChEBI. As expected, recall drops, but only by 0.3 to 1.9 percentage points.

**Table 6:** Performance (in %) of individual systems in combination with NP chunking, before and after stop-word removal.

	Baseline + NP chunking			Baseline + NP chunking + stop-words		
		
	Precision	Recall	F-score	Precision	Recall	F-score
ChEBI	56.3	39.4	46.4	77.5	38.5	51.5
ChEMBL	87.8	18.2	30.1	88.6	18.2	30.1
ChemSpider	70.1	37.9	49.2	81.5	37.3	51.2
DrugBank	62.9	16.8	26.5	76.6	16.7	27.5
HMDB	73.5	33.7	46.2	82.0	33.1	47.2
NPC	46.8	26.0	33.5	59.1	25.7	35.9
TTD	66.6	14.4	23.6	83.0	14.0	24.0
PubChem	32.7	57.0	41.6	61.5	55.9	58.6
Jochem	64.3	50.6	56.7	67.4	50.6	57.8
UMLS	36.6	49.2	42.0	44.3	48.9	46.5
ChEBI Family	18.4	15.9	17.1	28.8	15.6	20.3

The removal of stop-words in combination with the NP chunking system gives a further improvement of performance, but to a much smaller extent than for the baseline system. This is largely because most of the stop-words are not part of the noun phrases and disregarding them has no effect. Based on a comparison between the performances in Table 3 and Table 6 we decided to dispense with NP chunking as there was no gain.

### Regular expressions

The regular expressions detected 44.4% of the chemical database identifiers, with a precision of 90.4%. Further analysis of the false-positive and false-negative detections showed many partial extractions, e.g., "LY2090314" was extracted as an identifier while a prefix had also been annotated as part of the identifier ("[(14)C]LY2090314").

### Ensemble system

We evaluated different combinations of terminological resources (applying different case-sensitivity settings), grammar-based recognizers, and regular expressions on the training data. The ensemble system with the best F-score consisted of the combination of ChEBI, HMDB, LeadMine, and the regular expressions, yielding an F-score of 66.6% (Table 7).

**Table 7:** Performance of the ensemble system on the training material.

	CDI task			CEM task		
		
Ensemble system	Precision	Recall	F-score	Precision	Recall	F-score
ChEBI, HMDB, LeadMine, and RegEx	70.1	63.7	66.7	70.9	62.8	66.6
+ Missed terms added to dictionary	73.4	91.0	81.3	73.8	89.4	80.9
+ False-positive terms added to stop-word list	87.6	89.4	88.5	86.4	87.6	87.0
+ Removal of overlapping terms	91.8	89.1	90.9	91.8	87.4	89.5

The dictionaries performed best with case-sensitive matching but the differences with partial case-sensitive and with case-insensitive matching were marginal. Further addition of terminological resources to the ensemble system improved recall but decreased precision to a larger extent. For example, the addition of PubChem provided the largest increase in recall (about 7 percentage points), but decreased precision with about 8.9 percentage points, resulting in a drop in F-scores of 2.1 percentage points. Also note that the ensemble system had a better F-score than any of the individual systems (cf. Table 3). When we applied a voting approach, using all our sources and resources and varying the voting threshold between 1 and 15, the best F-score was 65.3% (precision 76.6%, recall 56.9%) for a threshold of 4.

We further analyzed the number of unique true positives (TPs) per entity type found by each of the systems within the ensemble system (Table 8). From a total of 37469 TPs captured by the ensemble system, 4139 cases were unique to ChEBI (mostly formula and abbreviation), 1878 were unique to HMDB (mostly trivial and abbreviation), 9480 cases were unique to LeadMine (mostly systematic terms) and 280 cases were unique to Regular expressions.

**Table 8:** Number of unique true positives found by each system in the ensemble system.

Entity type	Regex	LeadMine	CHEBI	HMDB
Trivial	8	1655	888	**711**
Systematic	0	**3945**	198	136
Family	0	2643	79	325
Formula	0	613	**1866**	110
Abbreviation	39	515	1093	596
Multiple	0	11	2	0
Identifier	**229**	98	13	0
Total	280	9480	4139	1878

We tried to further improve our system by expanding our dictionary with the gold-standard annotations from the training material that were missed by our system. This greatly improved the recall and F-score values (Table 7), although these estimates are optimistically biased since we evaluated the performance on the same dataset from which the newly added terms were derived. We also added all false-positive terms, i.e., terms indexed by our system but not annotated within the corpus (e.g., "peptide" and "carcinogen"), to our stop-word list, which further improved performance. Furthermore, we removed the shorter of two overlapping terms, which added 2.5 percentage points to the F-score, to reach 90.9% for the CDI task and 89.5% for the CEM task.

We submitted various runs to evaluate the system performance on the test set for both the CDI task and the CEM task (Table 9). The F-score of the baseline ensemble system improved by 9 percentage points after adding the false-negative terms of the training material to the dictionary and the false-positive terms to the stop-word list. A small further improvement was seen after the removal of overlapping terms, corroborating our findings on the training material. The best ensemble system obtained F-scores of 77.6% and 77.8% for the CDI and CEM tasks, respectively. Additional runs with a more recall-oriented system that included PubChem improved recall only slightly (about 3 percentage points) but greatly reduced precision (about 16 percentage points). We also tested whether removal of dictionary terms with low confidence scores would further improve the results, but this was not the case.

**Table 9:** Performance of the ensemble system on the test set.

	CDI task			CEM task		
		
Ensemble system	Precision	Recall	F-score	Precision	Recall	F-score
ChEBI, HMDB, LeadMine, and RegEx	71.5	64.8	68.0	73.1	64.6	68.6
+ Missed terms added & extended stop-word list	81.0	72.1	76.3	82.5	71.6	76.7
+ Removal of overlapping terms	84.6	71.7	77.6	85.8	71.2	77.8

## Discussion

Extracting chemical terms from unstructured text has proven to be a difficult task [3]. Here we present an ensemble approach that combines a grammar-based approach to capture systematic chemical identifiers with a dictionary-based approach and regular expressions to capture non-systematic names. The ensemble system performed better than any individual system. Stop-word removal was shown to greatly improve system performance, as did the addition of false-negative and false-positive terms from the training material to the dictionary and stop-word list, respectively. The effect of different types of case-sensitive matching, use of NP chunking, and removal of dictionary terms that were likely to be highly ambiguous or non-chemical, did not essentially change the performance.

Our initial assumption about the beneficial effect of NP chunking on compound recognition was only partially met, in that the use of NP chunking alone improved performance but there was no additional value in combination with stop-word removal (cf. table 6). In a previous study by Kang et al. [51] dictionary-based recognition of diseases in scientific abstracts was improved by employing NLP techniques, including NP chunking. However, in that study only a small stop-word list was used. Also, chunk recognition in disease-related abstracts may be easier than in chemical abstracts, which can contain complex chemical names with multiple punctuation marks (e.g., hyphens, brackets).

On the test set, our best ensemble system achieved F-scores of 78% for both challenge tasks. The results of our ensemble system on the training material are much better than on the test set (cf. Tables 7 and 9), but clearly this is due to the fact that we used the training data to improve the system. However, if we compare the baseline ensemble system, for which no training was needed, the F-scores on the training and test sets were almost similar for the CDI and CEM tasks.

From the 27 teams that participated in the BioCreative CHEMDNER challenge, 20 teams used machine-learning methods to extract chemical terms from text. The most frequently used method was CRF [8]. The best scoring system for the CDI subtask [52] managed to gain a precision of 87%, a recall of 89%, and an F-score of 88%. This system uses CRF along with word clustering to extract terms. The state of the art system for the CEM subtask [17] obtained 89% precision, 86% recall, and 87% F-score. This system also uses CRF along with several pre-processing steps to extract chemical terms from text. With an F-score that was about 10 percentage points lower than the best systems, our ensemble system ranked eighth for the CDI task and seventh for the CEM task. Tuning of the grammar-based systems that we considered, could have resulted in a higher F-score. For example, LeadMine also participated in the challenge as a separate software system [16]. After tuning, LeadMine achieved an F-score that was nine percentage points higher than our ensemble system, and 32 percentage points higher than the baseline LeadMine system that we used. Also ChemAxon participated in the challenge and obtained an F-score of 77% (an increase of 22 percentage points compared to the version we used). Among the teams who used lexical resources, ChEBI, PubChem and DrugBank were most often used; 13 teams also used a stop-world list. Irmer et al. [53] used a dictionary-based approach along with modules to recognize formulas or handle specific scenarios (such as abbreviation or acronym expansion) and obtained an F-score of 77%. They introduced a set of words in a so-called grey list. Terms in this list were only annotated in specific circumstances. Some systems (e.g. [54]) also tried to create an ensemble system by combining machine learning, dictionary-based approaches and regular expressions, but obtained lower F-scores than our ensemble system. Finally, in our approach the ensemble system merges the outputs of a selected set of individual systems. Our results indicate that this approach produced a better result than a simple voting scheme. However, we did not explore more sophisticated approaches, such as weighted voting or integration into a learning framework [55]. Application of these techniques may further improve the performance of an ensemble system.

Our approach has several advantages. First, use of the terminological resources and grammar-based recognizers did not have to be trained. This is an advantage over machine-learning approaches that require a large training set, which is laborious and expensive to create. On the other hand, our results also indicate that a substantial performance improvement can be gained by using the training data to expand the dictionary and the stop-word list. Thus, if training data are available, they can straightforwardly be used to improve system performance for both dictionary-based and grammar-based approaches.

A second advantage is that our system can provide structures for most of the found terms. Although the supply of information about structures was not required for the CHEMDNER tasks, chemists are generally interested in the chemical structure of a chemical identifier recognized in text. The terminological resources in the ensemble system (ChEBI and HMDB) contained MOL files, and also the grammar-based method (LeadMine) can provide structures for the extracted terms. Only the terms extracted with the regular expressions and terms that were added based on the training data, are not linked to structure information.

There are also several limitations. While the precision of our best ensemble system was an acceptable 86%, the recall was a more modest 71%. Including other dictionaries in the ensemble improved recall, but deteriorated precision to a much larger extent. Also, we noticed that many of the missed chemical terms were due to tokenization issues, e.g., the formulas "WC" and "Na" were missed in the context of "(nano-WC)" and "(I(Na))", respectively (PMID 22954532). Improvement of our tokenizer will further be investigated.

Another limitation of the current ensemble system is that some of the entity types were poorly recognized, in particular the entity types Multiple and Formulas. Terms of these types are not well covered in our dictionary. Better recognition may be possible by the use of regular expressions specifically developed for these types.

Finally, it should be noted that we used the grammar-based recognition tools with their default parameter settings, and did not try to tune them to the tasks at hand. Further improvements may be possible if such tuning were done.

## Conclusion

We developed an ensemble system that combines dictionary-based and grammar-based approaches to chemical named entity recognition, and obtained F-scores of 78% on the two CHEMDNER challenge tasks. The baseline version of the system did not require training, but we were readily able to improve performance by making use of the available training data. The system is capable of providing structure information for most of the compounds that are found. Improved tokenization and better recognition of specific entity types will likely further increase system performance.

## Competing interests

The authors declare that they have no competing interests.

## Authors' contributions

SA extracted and processed the data. SA, KH, EvdH, and EvM analyzed the data. JK supervised and coordinated the project. SA drafted the manuscript and KH, EvdH, EvM, and JK revised it. All authors read and approved the final manuscript.
